# Predictive Modeling of Drug Response in Non-Hodgkin’s Lymphoma

**DOI:** 10.1371/journal.pone.0129433

**Published:** 2015-06-10

**Authors:** Hermann B. Frieboes, Bryan R. Smith, Zhihui Wang, Masakatsu Kotsuma, Ken Ito, Armin Day, Benjamin Cahill, Colin Flinders, Shannon M. Mumenthaler, Parag Mallick, Eman Simbawa, A. S. AL-Fhaid, S. R. Mahmoud, Sanjiv S. Gambhir, Vittorio Cristini

**Affiliations:** 1 Department of Bioengineering, University of Louisville, Louisville, KY, 40202, United States of America; 2 James Graham Brown Cancer Center, University of Louisville, Louisville, KY, 40202, United States of America; 3 Department of Pathology, University of New Mexico, Albuquerque, NM, 87131, United States of America; 4 Molecular Imaging Program at Stanford (MIPS), Department of Radiology, Stanford University, Stanford, CA, 94305, United States of America; 5 Department of Biological Chemistry, University of California at Los Angeles, Los Angeles, CA, 90095, United States of America; 6 Center for Applied Molecular Medicine, University of Southern California, Los Angeles, CA, 90033, United States of America; 7 Department of Mathematics, Faculty of Science, King Abdulaziz University, Jeddah, 21589, Saudi Arabia; 8 Department of Bioengineering, Stanford University, Stanford, CA, 94305, United States of America; 9 Department of Materials Science & Engineering, and Bio-X, Stanford University, Stanford, CA, 94305, United States of America; 10 Department of Chemical Engineering and Center for Biomedical Engineering, University of New Mexico, Albuquerque, NM, 87131, United States of America; Institute of Clinical Physiology, c/o Toscana Life Sciences Foundation, ITALY

## Abstract

We combine mathematical modeling with experiments in living mice to quantify the relative roles of intrinsic cellular vs. tissue-scale physiological contributors to chemotherapy drug resistance, which are difficult to understand solely through experimentation. Experiments in cell culture and in mice with drug-sensitive (*Eµ-myc/Arf-/-*) and drug-resistant (*Eµ-myc/p53-/-*) lymphoma cell lines were conducted to calibrate and validate a mechanistic mathematical model. Inputs to inform the model include tumor drug transport characteristics, such as blood volume fraction, average geometric mean blood vessel radius, drug diffusion penetration distance, and drug response *in *cell culture. Model results show that the drug response in mice, represented by the fraction of dead tumor volume, can be reliably predicted from these inputs. Hence, a proof-of-principle for predictive quantification of lymphoma drug therapy was established based on both cellular and tissue-scale physiological contributions. We further demonstrate that, if the *in vitro* cytotoxic response of a specific cancer cell line under chemotherapy is known, the model is then able to predict the treatment efficacy *in vivo*. Lastly, tissue blood volume fraction was determined to be the most sensitive model parameter and a primary contributor to drug resistance.

## Introduction

Lymphoma drug response has predominantly been studied at the subcellular-to-cellular scale (molecular cancer biology), or at the whole-organ and/or systemic scale (oncology and clinical applications) (e.g., see recent studies [1–5]). Despite a multitude of genetic, proteomic, and histological analyses to understand lymphoma progression and treatment, drug resistance remains a major challenge to chemotherapy [6]. It is well known that specific phenomena at various physical scales contribute to this resistance. At the molecular scale, genetic/proteomic make-up can lead to intrinsic cellular drug resistance [7]; at an intermediate scale, transport barriers in the tissue microenvironment due to diffusion gradients of oxygen, nutrients, and drug may impede optimal response to cell-cycle specific drugs through cell quiescence as well as insufficient drug levels to achieve cytotoxicity [8, 9]; at the tissue scale, tumor morphological instability as a result of vascularization irregularities may lead to tumor tissue fragmentation and increased invasiveness [10].

The relative contribution to drug resistance due to intrinsic cellular mechanisms vs. physiologic resistance originating from the 3D tumor microenvironment is unclear. Cellular-intrinsic drug resistance depends on molecular mechanisms that support individual cell survival, including increased repair pathways and detoxification, adaptations leading to failure of apoptosis, and alterations in transmembrane drug transport [11]. These mechanisms define the resistant phenotype at the molecular scale primarily based on the cells’ molecular and genetic properties. Physiologic resistance that is not a direct result of the molecular phenotype can arise when a cell enters a quiescent proliferative state, thus becoming insensitive to the mode of action of cell-cycle specific drugs such as doxorubicin (Dox) and cyclophosphamide. An arrested proliferative state is a well-studied mechanism of physiological resistance to most chemotherapeutic agents currently in use. This resistance may depend on tumor and vascular geometry as well as distance to blood vessels and diffusion and pressure gradients.

Assessing drug response prior to treatment would help to obviate unnecessary, high-cost treatments and alleviate the high-morbidity of lymphoma. Toward this end, we develop a combined experimental and mathematical modeling approach to assess chemotherapy response in Non-Hodgkin’s Lymphoma. In previous work [12], we proposed a framework for modeling lymphoma growth in living subjects based on extensive calibration of model parameters from experimental data obtained from an *in vivo* mouse model. Here, we extend this work to model the drug response by using a general mechanistic modeling method we recently developed, which has been successfully applied to predicting patient-specific treatment outcome based on measurements from histopathological data [13] and to quantifying *in vitro* tumor response to both free and nano-carrier mediated drug delivery [14]. We then compare the simulated results to the response observed in the *in vivo* mouse system. A primary goal of this work is to offer a platform to generate and test hypotheses related to the complex interaction and contribution of intrinsic cell- and physiological tissue-scale characteristics to disease progression and chemotherapy response. Longer term, a coordinated effort to include clinical data may help improve strategies for lymphoma treatment in patients, especially for those with drug-resistant disease, e.g., by developing an *in silico* tool to systematically evaluate potential drug therapy outcomes to select optimal therapeutic strategies prior to actual treatment. Note that the modeling and associated considerations presented here also apply to *immunotherapy*, a rising promise in cancer treatment [15].

Although a number of theoretical models of tumor drug response have been developed in recent years (e.g., [16–24]), with some notable exceptions [25–29] very few have focused on lymphoma. In particular, Roesch et al. [29] studied the interactions between lymphoma and immune system cells during chemotherapy, while Maini and coworkers [27] modeled and analyzed outcomes for Non-Hodgkin’s lymphoma treated with Dox. Their model incorporated drug pharmacokinetics and pharmacodynamics, tumor cell-cycle kinetics, immature and mature vessels, and vascular structural adaptation, finding that treatment efficacy critically depended on the effects from heterogeneous blood flow modulated by the time interval between successive rounds of chemotherapy.

**Fig 1:**
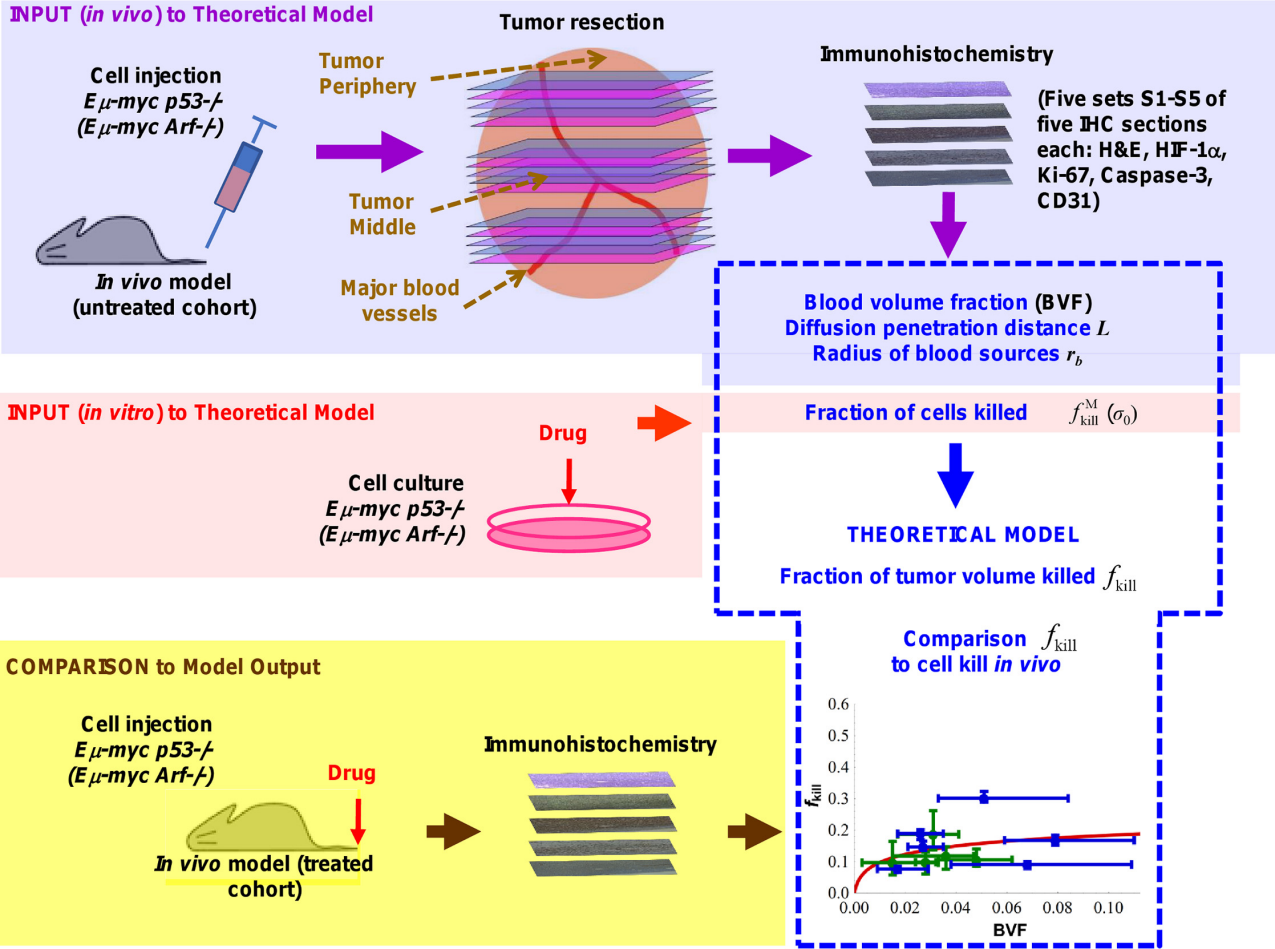
Strategy for model calibration and validation. Values for mathematical model input parameters are initially calibrated from experimental data obtained from untreated subjects and cell culture, yielding blood volume fraction, diffusion penetration distance, radius of blood sources, and fraction of cells killed in culture. Based on these parameter values, the model then calculates the fraction of tumor volume that would be killed *in vivo*, which can be compared to experimental data obtained from treated subjects.

Here, we model lymphoma drug response using a mathematical formulation that describes intra-tumor drug transport by linking cell- and tissue-scale biological phenomena. Our approach to constrain and inform the model relies on histopathological measurements of tumor characteristics that have been shown to be predictive of tumor growth [12] and response to drug treatment [18]. The strategy is described in **Fig 1**. Values for input parameters of the model are initially calibrated from data from untreated lymphoma tumor cells and tissue yielding blood volume fraction, diffusion penetration distance, radius of blood sources, and fraction of cells killed *in vitro*. Based on these parameters, the model calculates the fraction of dead tumor volume that would be expected *in vivo*. We compare the model predictions of treatment with results obtained in live mice. We use two types of lymphoma cells, i.e., drug-sensitive (*Eμ-myc/Arf-/-*) and drug-resistant (*Eμ-myc/p53-/-*) cells, which harbor loss-of-function of the alternative reading frame (ARF) protein (cellular response to oncogenic stress) along with the tumor suppressor 53 (*p53*) gene (DNA repair, growth arrest, and apoptosis), respectively. These cells were intravenously injected into C57BL/6 mice, leading to tumors that grow orthotopically within the inguinal lymph nodes [30]. Tumor slices obtained post-chemotherapy were stained for various cellular markers, including vascular endothelial cells and necrosis. The markers were quantified to enable a detailed comparison between drug-sensitive and drug-resistant tumors. By calibrating the model parameters from an initial set of experimental data obtained from untreated tumors, and then generating treatment results which are verified against cytotoxicity experiments *in vivo*, we are able to assess the relative roles of intrinsic cellular vs. physiological contributors to drug resistance and, further, to evaluate the model predictivity.

## Materials and Methods

### Cell Culture

*Eμ-myc/Arf-/-* and *Eμ-myc/p53-/-* lymphoma cells, harboring loss-of-function regions in the *Arf* and *p53* genes, respectively, were derived by intercrossing *Eμ-myc* transgenic mice with *Arf*-null and *p53*-null mice, all in the C57BL/6 background as described previously [31]. Cell lines were obtained from Dr. Scott Lowe's Laboratory [31] and authenticated, showing that the *Arf* and *p53* genes were deleted, respectively, and that these murine cells overexpress the *myc* gene. PCR was applied for detection using a specific primer. Western blotting was also used to confirm that either *Arf* or *p53* were respectively deleted in each cell line. Our experiments using these cell lines were performed in 2010 and 2011. The cells were cultured in 45% Dulbecco’s modified Eagle medium (DMEM) and 45% Iscove's Modified Dulbecco's Medium (IMDM) with 10% fetal bovine serum (FBS) and 1% penicillin G-streptomycin onto the feeder cells – Mouse Embryonic Fibroblasts (MEFs). The MEF cells were also obtained from Dr. Lowe’s laboratory [31].

### Cytotoxicity Experiments in Cell Culture

2,000 mouse embryonic fibroblast (MEF) cells were seeded per well in a 96-well plate, and then one day later *Eμ-myc Arf-/-* and *Eμ-myc/p53-/-* lymphoma cells (1,500/well) were spread onto the plate. Dox was added one hour later (top concentration: 10 μM and serially diluted 3X) and the cells were incubated at 37°C in 5% CO2 for 48h. The growth inhibition and corresponding IC50 were measured with the CellQuanti-MTT cell counting kit (BioAssay Systems, Hayward, CA).

### Murine Lymphoma Model

The *Eμ-myc* transgenic mouse model expresses the *Myc* oncogene in the B cell compartment, resulting in mice with transplantable B cell lymphomas. We chose this mouse model because it captures genetic and pathological features of the human disease and, given the appropriate genetic mutation, enables comparison of drug-resistant and drug-sensitive tumors [31, 32]. C57BL/6 mice were purchased from Charles River Laboratories (Wilmington, Massachusetts). All animal studies were approved by The Stanford University Institutional Animal Care and Use Committee. 1.0 × 10^6^ *Eμ-myc/Arf-/-* and *Eμ-myc/p53-/-* lymphoma cells were diluted with 200 μl of PBS and injected intravenously via the tail vein as previously described [31]. Intravital microscopy and macroscopic tumor observations were obtained for at least n = 4 mice per tumor group. The condition of the animals after injection was monitored 3–4 times per week.

### Cytotoxicity Experiments in Living Mice

Mice were injected via the tail vein on day 19 post-injection of lymphoma cells with 1 mg/mL (1.72 mM) Dox based on 10 mg Dox/kg bodyweight. This represents the maximum tolerated dose (MTD) for a typical mouse. This drug amount yields ~2.16 μM Dox concentration in the blood volume based on a typical mouse bodyweight of 20–25 g, and represents an upper limit for the concentration in the tumor volume (in comparison, the typical dose of 104 mg for an average human adult yields ~6.9 mM Dox concentration in the blood volume). Tumors were isolated similar to previous work [12, 30] on day 21 (2 days after Dox administration). 1% to 2% inhaled isoflurane was used for anesthesia. A heating plate was used to help recover body temperature prior to ambulation. No analgesics were indicated. The mice were sacrificed with CO2.

### Immunohistochemistry

*Eμ-myc/Arf-/-* and *Eμ-myc/p53-/-* driven tumors were excised from the inguinal lymph node area; at this stage, tumors were ~4–6 mm in lateral diameter. Tissues were fixed and paraffin-embedded. Five 2-μm thick sections were cut, within 5 μm of each other, in order to stain for histology markers and to be able to co-localize them in adjacent sections across the whole tumor. Sets of sections were then obtained every 100-μm along the tumor, with *Eμ-myc/p53-/-* (drug resistant) tumors yielding six sets of sections while *Eμ-myc/Arf-/-* (drug-sensitive) tumors, shrinking during treatment, yielded five sets of sections. The sections were de-paraffinized and rehydrated in PBS, and submitted to immunohistochemical (IHC) identification of cell viability (H&E staining), hypoxia (HIF-1α), vascularization (CD31), proliferation (Ki-67), and apoptosis (Caspase-3). For IHC, sections were incubated at 4°C with the primary antibody overnight: rabbit anti-mouse HIF-1α antibody (Abcam, Santa Cruz, CA), rabbit anti-mouse Ki-67 antibody (Labvision, Fremont, CA), rabbit anti-mouse Caspase-3 antibody (Cell Signaling Technology, Beverly, CA), and rat anti-mouse CD31 antibody (BD Pharmingen, San Diego, CA), then incubated for 1 hour at room temperature with a peroxidase-conjugated secondary antibody. Using digital pathology, samples were scanned and each complete section was stitched together with a Hamamatsu NanoZoomer 2.0RS (Shizuoka, Japan) at ×20 magnification.

### Mathematical Model of Drug Response

We recently developed a mathematical model [13] based on drug diffusion and perfusion to predict chemotherapy outcome, and successfully validated the model using data from colorectal cancer metastatic to liver and glioblastoma patients. Briefly, local drug concentration within the tumor is described using a diffusion-reaction equation that accounts for the diffusion and uptake of drug by tumor cells after extravasation:

$$\frac{1}{r}\frac{\partial }{\partial r}\left(r\frac{\partial \sigma }{\partial r}\right)-\frac{\sigma }{L^{2}}=0,$$ (1)

where *σ* is the local concentration of drug, *r* the radial coordinate scaled with the drug diffusion penetration distance $L=\sqrt{D/\lambda }$, *D* the diffusion constant of the drug, and *λ* the cellular uptake rate of drug (with a unit of inverse time). Solving Eq 1 for a single, straight cylindrical blood vessel radius *r*_b_ and integrating over the surrounding tissue domain, we obtain the fraction of tumor volume killed *f*_kill_ in closed form:

$$f_{\text{kill}}=f_{\text{kill}}^{\text{M}}(\sigma_{0})\cdot \text{BVF}\cdot \frac{2\cdot \sqrt{\text{BVF}}\cdot K_{1}(r_{\text{b}}/L)-2\cdot K_{1}((r_{\text{b}}/L)/\sqrt{\text{BVF}}))}{\sqrt{\text{BVF}}\cdot (r_{\text{b}}/L)\cdot K_{0}(r_{\text{b}}/L)\cdot (1-\text{BVF})},$$ (2)

where *K*_0_ and *K*_1_ are modified Bessel functions of the second kind of orders 0 and 1, respectively (see [13] for model derivation in detail). We note particularly that *f*_kill_ by intravenous drug administration is quantified as a function of a limited set of tumor-specific parameters, including the vascular density or blood volume fraction (BVF), *L*, *r*_b_, and fraction of cells killed *in vitro* $f_{\text{kill}}^{\text{M}}(\sigma_{0})$, which can all be directly measured from histopathology and monolayer cell culture experiments.

### Model Prediction

The model predictions were compared to measurements of tumor kill from histopathology images of treated tumors. The model inputs can thus be measured *in vivo* prior to treatment and be used to predict individual response. Since the tumor and histology sectioning were assumed to be isotropic, the predicted fraction of tumor volume killed can be directly compared to the fraction of tumor area killed measured from the histopathology data. Note that the basic concept of the model has also been validated across different cancer types (e.g., breast cancer [33] and pancreatic cancer [34]) as well as different treatment methods (e.g., immunotherapy [15] and nanomedicine [14]).

## Results

We first performed *in vitro* and *in vivo* experiments to obtain measurements for the mathematical model parameters. The *in vitro* experiments evaluated cytotoxicity of the drug-sensitive and drug-resistant lymphoma cells in culture, while the *in vivo* experiments evaluated tumors with these cells grown in live mice as described in **Methods**. Histology analysis of the tissue obtained *in vivo* provided insight into the lymphoma tissue characteristics. The mathematical model parameters were measured from the histology and *in vitro* data, and then input into the model to predict the drug response *in vivo*. Finally, a sensitivity analysis was performed to determine the relative significance of the main model parameters.

### Drug Response in Cell Culture

**Fig 2:**
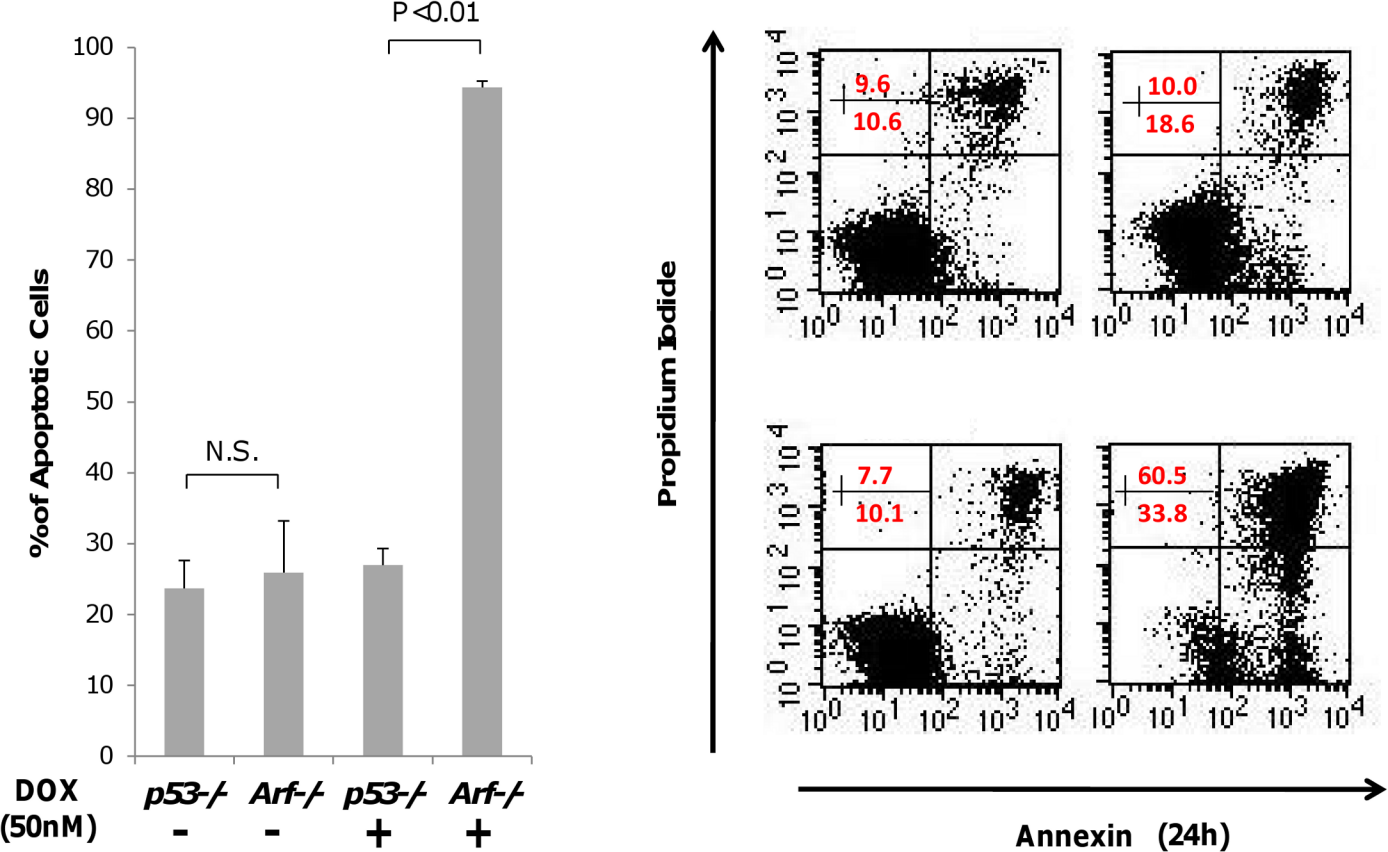
Drug response experiments *in vitro*. (*Left*) Measurement of *in vitro* cell kill in cell culture for *Eμ-myc/Arf-/-* and *Eμ-myc/p53-/-* cells after 48 hours at 50 nM Dox concentration (N.S.: not statistically significant). (Right) Results from a flow cytometry study were used to measure apoptotic cells. *Eμ-myc/p53-/-* cells are displayed along the top row with *Eμ-myc/Arf-/-* cells along the bottom; controls (no drug) are in the left column, and drug-treated cells (Dox) are in the right column. For each block, lower left quadrant represents live (proliferating) cells; lower right quadrant shows apoptotic cells; upper right quadrant shows dead cells.

The inhibitory concentration of Dox to achieve 50% growth inhibition (i.e., the “IC50”) was measured at 48 hours to be 3.5 nM for *Eμ-myc/Arf-/-* and 46.2 nM for *Eμ-myc/p53-/-* cells, indicating ~13× differential in drug response between the two cell lines at this drug concentration (**Table 1**). These results were confirmed in separate experiments at one of our laboratories (Mallick). In comparison to other cell lines, we measured 41.5 nM and ~1000 nM Dox IC50 for the well-characterized Jurkat (human acute T-cell leukemia) and Daudi (human B-cell Burkitt’s lymphoma) cell lines (**Table 1**). Under treatment, *Eμ-myc/Arf-/-*cells had a 3× higher apoptotic fraction compared to *Eμ-myc/p53-/-* cells (**Fig 2**, *left*; *P* < 0.01 for student’s *t*-test with α = 0.05). In comparison, cells without these mutations had no statistically significant difference in apoptosis.

**Table 1:** Doxorubicin (Dox) IC50 for murine *Eμ-myc/Arf-/-* and *Eμ-myc/p53-/-* lymphoma cells used in this study compared to well characterized Daudi and Jurkat cell lines.

Cell line	Dox (nM)	Origin
*Eμ-myc/Arf-/-*	46.2	Mouse Non-Hodgkin’s lymphoma
*Eμ-myc/p53-/-*	3.5	Mouse Non-Hodgkin’s lymphoma
Daudi	>1000	Human B-cell Burkitt’s lymphoma
Jurkat	41.5	Human acute T cell leukemia

Flow cytometry was used to evaluate propidium iodide (PI) versus cell apoptosis (Annexin V) for both untreated (control) and treated cells. The results show that whereas *Eμ-myc/Arf-/-* cells substantially decreased proliferation and increased apoptosis under drug exposure, the *Eμ-myc/p53-/-* cells remained relatively unaffected under treatment (**Fig 2**, *right*). The drug resistance differential between the *Eμ-myc/Arf-/-*and the *Eμ-myc/p53-/-* cells was at least one order of magnitude in both cases. These results reflect the intrinsic molecular-based resistance of the *Eμ-myc/p53-/-* cells compared to *Eμ-myc/Arf-/-*cells and further confirm the cell culture observations of drug resistance (**Table 1**).

### Evaluation of Lymphoma Characteristics

**Table 2**shows average values for necrosis, apoptosis, blood volume fraction, and hypoxia measured from IHC in the sections cut across untreated [12] and treated lymphoma tumors grown in the inguinal lymph node of the mice. The values were obtained by averaging the measurements for all the same-stained sections for each tumor type (see **Methods**). **S1 Fig** shows an example of a single measurement for blood volume fraction in the center of a treated tumor (Set S3). The percentage of stained tissue was obtained by calculating the ratio of stained to (stained + unstained) tissue. The other measurements were similarly obtained.

**Table 2:** Average of tumor measurements from IHC used for model calibration.

	Treated tumors		Untreated tumors	
Average	Drug sensitive	Drug resistant	Drug sensitive	Drug resistant
Necrosis	12.1% ± 5%	4.7% ± 3%	0.5% ± 0%	0.8% ± 1%
Apoptosis	3.8% ± 2%	5.1% ± 3%	4.8% ± 2%	20.7% ± 12%
Vessels (*)	3.1% ± 2%	4.5% ± 3%	1.8% ± 1%	2.8% ± 2%
Hypoxia	13.3% ± 8%	13.4% ± 6%	1.7% ± 1%	3.4% ± 4%
Proliferation	73.6% ± 13%	87.0% ± 6%	77.2% ± 18%	75.3% ± 14%

Vessels (*) are ~90% capillaries (10 μm in diameter) and ~10% veins (20 μm in diameter); this percentage was used to estimate the BVF. Note that the drug diffusion distance (40 ± 20 μm) is estimated in the best case not to exceed half that of O_2_ (based on how far hypoxic regions were measured away from the vessels).

The necrotic fraction for *Eμ-myc/Arf-/-* cells in the treated tumors was ~24× that of the untreated case, while this same fraction was ~5× that of the untreated case for the *Eμ-myc/p53-/-* cells, implying that the bulk of necrosis was drug-induced. Interestingly, the apoptotic fraction for the untreated *Eμ-myc/p53-/-* tumors was ~4× that of the treated cases. For both cell types, the blood volume fraction was comparable between the two types. Similarly, hypoxia was also comparable between the two cell types and increased under treatment ~6× for the *Eμ-myc/Arf-/-*and ~4× for the *Eμ-myc/p53-/-* cells, probably due to cytotoxicity of proliferating endothelial cells.

**Fig 3:**
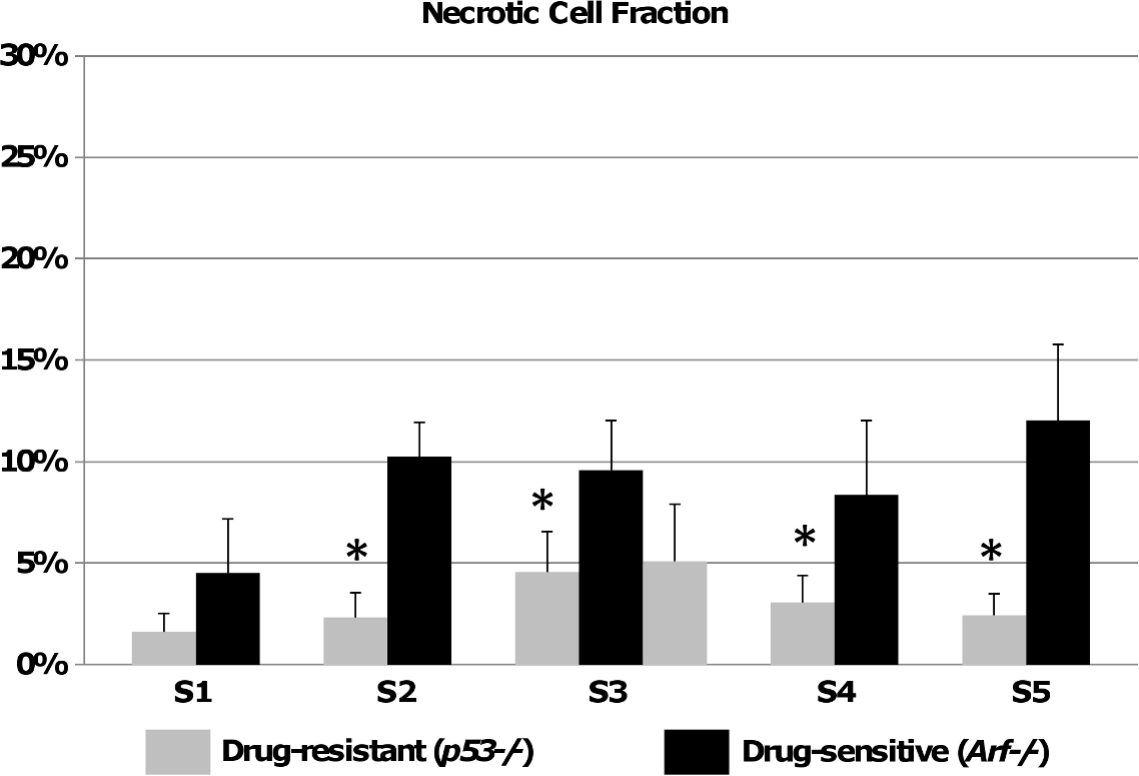
Necrotic cell fraction in murine lymphoma tumors after treatment with Dox. Data are shown for tumor slices S1 through S5. Most of the necrosis is a result of the drug treatment since necrosis measured in untreated tumors was negligible (**Table 2**). Note that the drug-sensitive tumors shrank in size after treatment and thus had one less histological slice than the drug-resistant tumors (to account for this, two slices of the drug-resistant tumor are included in the central region S3, i.e., five total slices for *Eμ-myc/Arf-/-* and six for *Eμ-myc/p53-/-*). All error bars represent standard deviation from at least n = 3 measurements in each section. Asterisks show level of statistical significance determined by student’s *t*-test with α = 0.05 (asterisk, *P* < 0.05).

The distribution of necrosis during treatment (**Fig 3**) shows that *Eμ-myc/p53-/-* cells in the tumor periphery (Sets S1 and S5, gray bars) were relatively unaffected by the drug, with most of the death occurring in the center of the tumor (Set S3, gray bars). In contrast, *Eμ-myc/Arf-/-*cells showed relatively uniform cell kill across the whole tumor (Set S1 through S5, black bars). Necrosis for the drug-sensitive cells was higher throughout most of the tumor except for the middle (Set S3) compared to the drug-resistant tumors, with a corresponding statistically significant decrease in cell density (*P* < 0.05; student’s t-test with α = 0.05). The necrosis distribution and magnitude correspond with the observation that the *Eμ-myc/Arf-/-* cells seem to be less dense in the middle of the lymph node compared to the *Eμ-myc/p53-/-* cells, in agreement with previous results [12]. This suggests a steeper diffusion gradient of drug and cell substrates (e.g., oxygen and nutrients) for the *Eμ-myc/p53-/-* from the middle (Set S3) to the periphery (Sets S1 and S5) based on the lymph node anatomy in which the arteries enter the node in the middle. Representative histology sections stained for cell viability (H&E), vascularization (CD31), and hypoxia (HIF-1α) are shown in **S2 Fig** and **S3 Fig**. The sections highlight the tighter packing of the drug-resistant *Eμ-myc/p53-/-* cells compared to the drug-sensitive *Eμ-myc/Arf-/-* in the samples taken in the middle of the tumor (**S3C and S3D Fig**).

**Fig 4:**
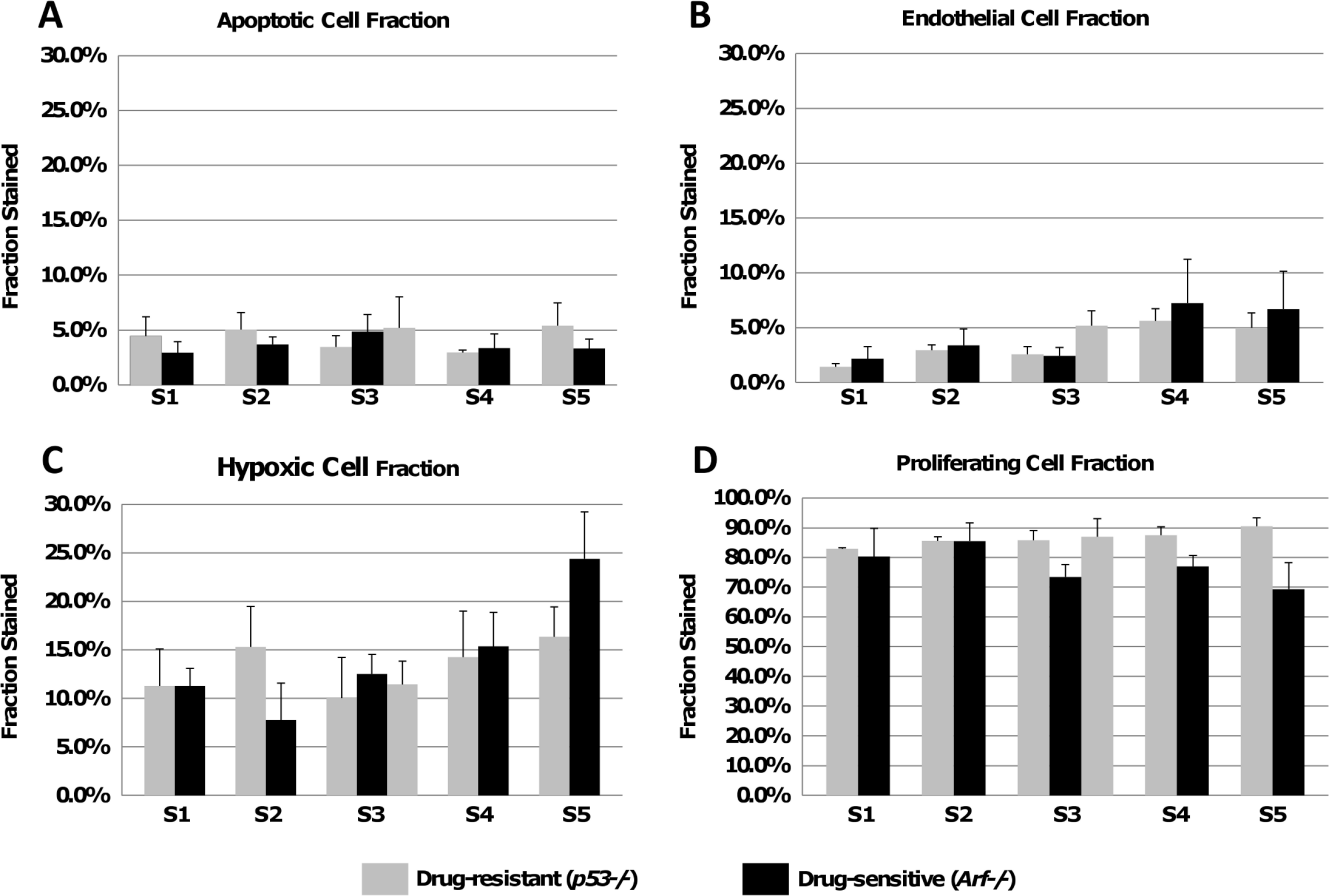
Whole-tumor measurement of lymphoma characteristics. Measurements from the IHC data after treatment with Dox shows cell fractions for: (**A**) apoptosis, (**B**) endothelium, (**C**) hypoxia, (**D**) proliferation. Note that the drug-sensitive tumors shrank in size after treatment and thus had one less histological slice than the drug-resistant tumors in the middle Set (S3). Error bars represent standard deviation (n = 3 regions of interest per slice).

**Fig 4**shows the measurements obtained from sets of histology sections across whole tumors post-treatment by identifying regions of interest (ROI; n = 3) in each section. In particular, the distribution of hypoxia (as measured by HIF-1α) for both cell types appears heterogeneous, with an increase towards the peripheral Sets S5/S6. We noted that most of the vessels were arteries, with veins being ~10% of all the vessels (based on cross-sectional area fraction).

### Measurements of Model Parameters

Three measurements of parameter values were obtained for each histology section in each set. The BVF was estimated as a percentage (**Table 2**) from the endothelial cell staining (CD31) by measuring the proportion of stained vs. unstained tissue, assuming that the BVF is proportional to the vascular density. **S1 Fig** shows representative sections stained for CD31, with accompanying percentages calculated. The diffusion penetration distance *L* was assumed in the best case not to exceed half that of O_2_, based on previous studies [35]; it is a reasonable assumption based on the larger size of the drug molecules. This was measured from the hypoxia (HIF-1α) staining as the distance from vessel cross-sections surrounded by viable tissue to the areas where staining was detected, yielding *L* = 40 ± 20 μm [12]. The blood source radius *r*_b_ was obtained from the endothelial cell staining (CD31) by taking the average of two orthogonal vessel cross-section measurements, yielding *r*_b_ = 5 ± 2 μm.

### Prediction of Drug Response

Corresponding to the sets of sections obtained from the excised tumors, there were 5 data measurements for the drug-sensitive *Eμ-myc/Arf-/-* cell line and 6 data measurements for the drug-resistant *Eμ-myc/p53-/-* cell line representing the percentage of tissue stained for each particular marker including necrosis, apoptosis, and vascular density. From the cell culture experiments *in vitro* (**Fig 2A**), we observe that the fraction of cell death from drug in the drug-sensitive cells was 3.5 times that of the drug-resistant cells. This was the observed ratio of the drug-sensitive cell line to drug-resistant cell line in drug response *in vitro*. Using this *in vitro* result as an input to the model, we then sought to predict drug response *in vivo*. Specifically, we rescaled the 6 data points for the drug-resistant cell line by 3.5 and then combined all the data points for both cell lines to evaluate the model’s predictivity. A regression analysis and least-squares fitting of Eq 2 was performed using the Mathematica routine “NonlinearModelFit” [36] applied to the measured tumor kill *f*_kill_ and blood volume fraction BVF. This resulted in estimates of two parameters *r*_b_ / *L* and $f_{\text{kill}}^{\text{M}}$, which produced the best fit.

**Fig 5:**
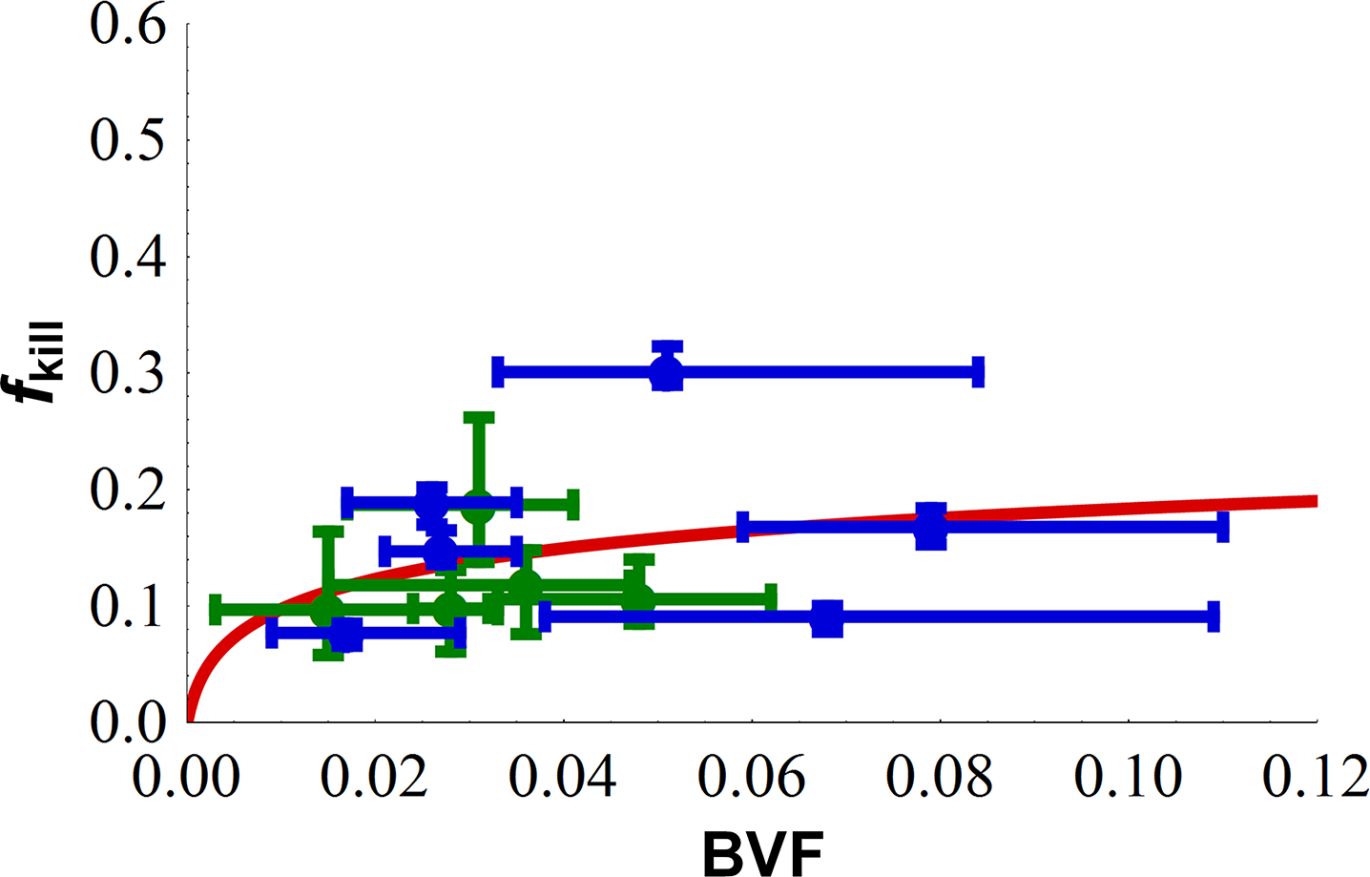
Mathematical model predicts lymphoma tumor death due to chemotherapy drug treatment. Comparison of histopathology measurements with mathematical model predictions (Eq 2, solid lines) based on estimates of two parameters *r*_*b*_ / *L* and $f_{\text{kill}}^{\text{M}}$. Data points for drug-resistant cells (blue) were scaled by 3.5 (see **Fig 2A**) to be comparable with data for drug-sensitive cells (green). Obtained *R* ^2^ = 0.86; estimated $f_{\text{kill}}^{\text{M}}$ = 0.25, and *r*_*b*_ / *L* = 0.068. Diffusion distance of drug from the vessels (40 ± 20 μm) was assumed in the best case not to exceed half that of O_2_. Each point represents measurements from one tumor Set; 5 data points for the drug-sensitive cell line (green) and 6 data points for the drug-resistant cell line (blue).

**Fig 5**shows the results of the fraction of tumor volume killed *f*_kill_ from chemotherapy as a function of BVF predicted by the model compared to those measured from the histopathological samples of drug-sensitive and drug-resistant tumors *in vivo*. The model’s coefficient of determination *R* ^2^ between the observed data and model (predicted) result was 0.86, and hence was considered acceptable in explaining the relationship between $f_{\text{kill}}^{\text{M}}$ and input parameters BVF and *r*_*b*_ / *L* (also see **S4 Fig** for how model predictions change with values of $f_{\text{kill}}^{\text{M}}$ and *r*_*b*_ / *L* other than their best fits). However, we note that there exists noticeable variance in experimental measurements of BVF and dead tumor area for each tissue section (n = 3 data points per section). This variance reflects the heterogeneity in tumor physical properties [8, 9] which may lead to non-uniform drug penetration and tumor response.

### Significance of Model Parameters

**Fig 6:**
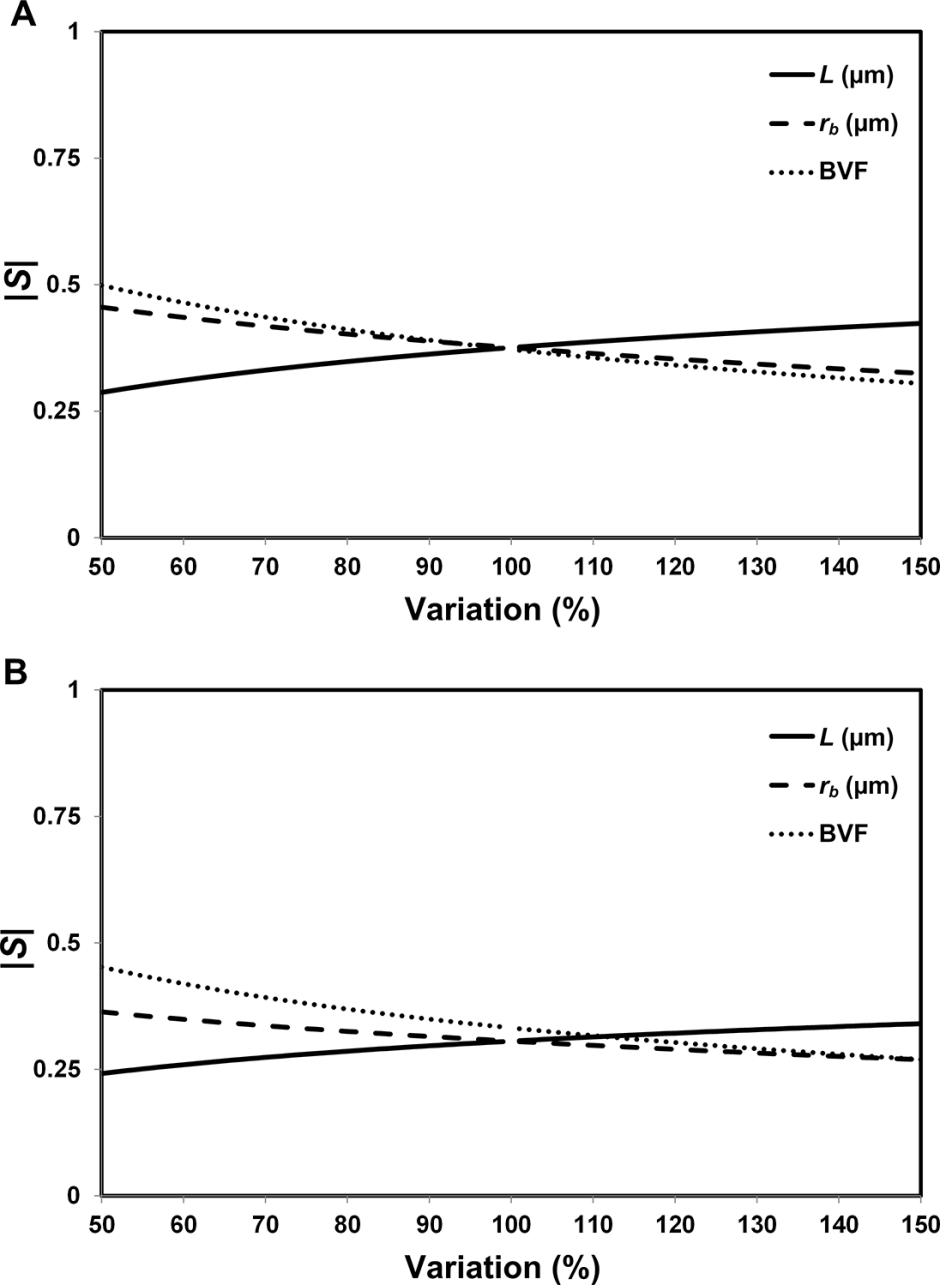
Sensitivity analysis results. Plots of absolute values of sensitivity coefficients for the three parameters for **(A)** the drug-sensitive cell line, *Eμ-myc/Arf-/-* and **(B)** the drug-resistant cell line, *Eμ-myc/p53-/-*. The range of variation for each parameter is listed in **S1 Table**. *S* represents sensitivity coefficient.

We performed a sensitivity analysis (**S1 Table**) to determine the relative effect of model parameters on *f*_kill_ for both drug-sensitive and drug-resistant cell lines, using a single-parameter-variation method [37–39]. The sensitivity coefficient *S* (**S1 Text**) was used to ascertain the impact of these individual parameter perturbations on the system output. In brief, the larger the absolute value of *S*, the more sensitive the system output is to the particular parameter under consideration. Here, our analysis focused on three of the four model parameters: diffusion penetration distance *L*, average tumor blood vessel radius *r*_b_, and tumor blood volume fraction BVF. The fourth parameter $f_{\text{kill}}^{\text{M}}$ is a multiplying factor of the model (Eq 2), i.e., the model output varies linearly with this parameter leading to a sensitivity coefficient *S* of 1 regardless of the degree of perturbation; thus, this parameter was not considered. The results are shown in **Fig 6**. For both cell lines, we find that the model output was most sensitive to BVF, followed by *r*_b_ and *L*. This analysis confirms that the tumor’s degree of vascularization has a dominant impact on the efficiency of drug delivery and hence the fraction of tumor killed by the drug treatment. The output of Eq 2 thus serves to *predictively quantify* the non-linear relationship between treatment outcome and the tumor’s degree of vascularization.

## Discussion

In order to help elucidate the relative contributions to drug resistance by intrinsic cellular mechanisms vs. tumor physiological characteristics, we apply a mathematical model to predict drug response in living subjects. The model predicts the overall fraction of tumor volume killed based on parameters that can be directly obtained from histopathology and cell culture cytotoxicity data prior to treatment. Cytotoxicity data in cell culture (**Fig 2**) fail to provide insight into the dependence of tumor response *in vivo* in living mice (**Fig 3**) on the microenvironment and associated physical transport barriers. While this is well-known, we show that a mathematical model is able to quantitatively capture the effects of heterogeneous tumor under-vascularization (**Fig 5**) using physiological measurements of untreated tumors, indicating that tumor kill in living subjects can be quantified based on these phenomena. In particular, diffusion of drug from the central vessels within the lymphoma may affect drug efficacy in the tumor peripheral regions, as shown by measurements of necrotic cell fraction for the drug-resistant tumors being higher in the center and lower on the periphery (**Fig 3**). This is consistent with our previous observations of higher cell density for drug-resistant lymphoma [12], implying the existence of steeper diffusion gradients not only due to under-vascularization but also due to higher tissue density. Interestingly, the necrotic cell fraction in the central tumor region (Set S3) is of comparable value between the two cell types, suggesting comparable drug efficacy when the diffusion barrier is minimized.

Cellular stress as a result of under-vascularization can severely affect the drug response. We have previously simulated the effect of the tumor microenvironment including under-vascularization on tissue morphology [10, 40–43]. The results suggest that marginally stable oxygen and nutrient conditions could directly affect tumor morphological stability and offer a challenge to drug therapy by promoting tumor cell invasiveness and leading to complex infiltrative tumor morphologies (as observed *in vitro* [44]), depending on the magnitude of cell adhesion forces that maintain non-invasive, more compact tumors. The combination of higher cell packing density with steeper diffusion gradients may increase drug resistance synergistically, as we have previously observed [18] and simulated [23], with a higher IC50 for more compact, drug-resistant tumors. Synergism may be due to increased drug binding to ECM in tumor areas proximal to the vasculature, while penetration of oxygen and cell nutrients as well as drug to areas distal to vessels is significantly reduced due to higher cell packing, hence exacerbating the resistance effect of diffusion gradients.

The mathematical model was shown (among the three parameters under consideration, i.e., *L*, *r*_b_, and BVF) to be most sensitive to BVF for both cell lines. This particularly suggests that, for cancer cells resistant to therapies, BVF may be a more valuable therapeutic target than the other parameters in improving the treatment effect (i.e., in increasing *f*_kill_). This result is consistent with pioneering experimental [45, 46] and modeling [10, 41] observations that angiogenesis inhibitors, which lower the BVF, may actually lead to increased drug resistance and thus could help explain why these inhibitors have not been as successful as originally hoped in controlling cancer growth [47]. The sensitivity analysis can further aid in guiding experiments to further test this hypothesis and potentially provide insight into the development of novel treatment modalities that exploit tumor vascular characteristics and could help predict appropriate drug combinations. We note that lymphoma blood vessels are not necessarily straight cylinders as assumed by the mathematical model. A tortuous vascular representation would be expected to exacerbate heterogeneity in the simulated fraction of tumor volume killed. Further, histology sections are not necessarily representative of the complex 3D tumor microenvironment, e.g., they may not account for diffusion from vessels above or below the sections. Also, the variability in parameter values between sets of histologic sections is unknown, although we assume that the variations are continuous from one section to the next; as we have previously observed via intravital microscopy (IVM) using a window chamber and mouse tumor model; there are no abrupt changes in the histological data. Further, we assume that CD31 staining corresponds to functional blood vasculature, although this staining may also include lymph as well as non-functional vessels. A more precise identification of functional blood vasculature will be pursued in the future through IVM experiments in living mice.

The model of lymphoma growth previously presented [12], which is based on modeling diffusive transport through tumor tissue, is implicitly contained in the model of drug response applied in this study. We envision that this integrated experimental and modeling approach will enable the prediction of drug response for specific tumors based on information collected from biopsy, e.g., blood volume fraction (BVF), diffusion penetration distance *L*, blood source radius *r*_b_, and fraction of cells killed from cytotoxicity assays *in vitro* $f_{\text{kill}}^{\text{M}}$. BVF could also be obtained using IVM methods [48] or other imaging techniques such as nuclear imaging or magnetic resonance imaging [49]. From this information, the fraction of tumor volume killed *f*_kill_ would be predicted based on different drug concentrations achieving various cytotoxic effects in cell culture. The area-under-the-curve based on these drug concentrations can be scaled to the patient to determine the corresponding plasma concentration. The dosing frequency of drug to achieve optimal tumor remission can then be calculated by simulating the vascular concentration after each bolus of drug. Further, the model could also be used to help estimate potentially synergistic effects produced by multiple drugs.

Current paradigms for lymphoma treatment are generally inadequate in that they may not take advantage of patient-specific tumor information [50]. The types of drugs to use and the strategy for their administration may be difficult to optimize in order to minimize drug resistance. A more quantitative, predictive evaluation of chemotherapy response before treatment would allow tailoring to individual patients’ tumors with the goal to overcome drug resistance. This would help improve outcomes by potentially identifying optimal drug regimens and administration protocols. Since the functional relationships in the model are mathematical formulations of biological hypotheses, this approach also enables the development of novel hypotheses that could be tested experimentally. Here, we have integrated experimental data with mathematical modeling to accurately predict the tumor response to a chemotherapeutic in a murine model of lymphoma. While this information helps to quantitatively link drug-sensitive and drug-resistant cell phenotypes to tissue-scale drug response, the ultimate goal is for such an integrated computational/experimental approach to help evaluate proposed chemotherapy in humans based on a small set of tumor-specific input parameters.

## Supporting Information

S1 Table Parameter values. Reference (standard) values used in the local sensitivity analysis for both drug-sensitive and drug-resistant cell lines.
(DOCX)

S1 Text Supplementary material. (DOCX)

S1 Fig Example of measurement of model parameters from the histology data. The process is illustrated through the quantification of CD31 staining in Set S3 in the center of the tumor reflecting blood volume fraction for *Eμ-myc Arf-/-* (drug-sensitive) and *Eμ-myc p53-/-* (drug-resistant) lymphoma cells. Positive staining shown in panels A and B is converted to red and negative staining to green in panels C and D to obtain a quantitative measure of apoptotic activity, as calculated in the text. Results are shown in bottom right insets.
(TIF)

S2 Fig Histology data. Representative whole-tumor histology sections for *Eμ-myc/Arf-/-* (left column) and *Eμ-myc/p53-/-* (right column) tumors, showing viable and necrotic cells (stained for H&E, panels A and B) and hypoxia (stained for HIF-1α, panels C and D, brown color) in the middle of the tumor (Set S3). Bar, 2 mm.
(TIF)

S3 Fig Vascularization data. Representative whole-tumor vascularization (CD31) staining for *Eμ-myc/Arf-/-* (A) and *Eμ-myc/p53-/-* (B) tumors. Higher magnification (100x) images (C & D) show corresponding typical vessels (brown color) (100x). Capillaries are thinner elongated structures while veins are larger. The tighter packing of the drug-resistant *Eμ-myc/p53-/-* cells compared to the drug-sensitive *Eμ-myc/Arf-/-* can be visually appreciated in these samples taken in the middle of the tumor (Set S3). (40x).
(TIF)

S4 FigModel predictions change with varying $f_{\text{kill}}^{\text{M}}$ and *r*_*b*_ / *L* values other than their best fits.The bold solid line in red represents the best fit case determined in **Fig 5**. Color scheme: {$f_{\text{kill}}^{\text{M}}$ = [0.5, 1.0, 1.5]-fold of its best fit} = {gray, red, gray}; green for drug sensitive cell line (*Eμ-myc/Arf-/-*) and blue for drug resistant cell line (*Eμ-myc/p53-/-*). Line scheme: {*r*_*b*_ / *L* = [0.5, 1.0, 1.5]-fold of its best fit} = {dashed, solid, dotted}.(TIF)
