# Supplementary material for: Predictive Modeling of Drug Response in Non-Hodgkin’s Lymphoma
Source: PLoS One. 2015 Jun 10;10(6):e0129433. doi: 10.1371/journal.pone.0129433 (PMC4464754; doi:10.1371/journal.pone.0129433)
Supplement: S1 Table — Reference (standard) values used in the local sensitivity analysis for both drug-sensitive and drug-resistant cell lines. (DOCX) [file pone.0129433.s001.docx]

**Table S1.** Parameter reference values used in the local sensitivity analysis, for both drug-sensitive and drug-resistant cell lines.

**Part I:** For drug-sensitive cell line, *Eµ-myc/Arf-/-*.

| Parameter | Reference value | Source | Variation range | Max \|*S*\| | Variation |
| --- | --- | --- | --- | --- | --- |
| *L* (μm) | 40 | Histopathological image measurements | [20−60] | 0.424 | 1.5-fold |
| $r_{\text{b}}$ (μm) | 5 | Histopathological image measurements | [2.5−7.5] | 0.456 | 0.5-fold |
| BVF | 0.031 | Histopathological image measurements | [0.0155−0.0465] | 0.499 | 0.5-fold |

**Part II:** For drug-resistant cell line, *Eµ-myc/p53-/-*.

| Parameter | Reference value | Source | Variation range | Max \|*S*\| | Variation |
| --- | --- | --- | --- | --- | --- |
| *L* (μm) | 40 | Histopathological image measurements | [20−60] | 0.340 | 1.5-fold |
| $r_{\text{b}}$ (μm) | 5 | Histopathological image measurements | [2.5−7.5] | 0.363 | 0.5-fold |
| BVF | 0.045 | Histopathological image measurements | [0.0225−0.0675] | 0.452 | 0.5-fold |
