# Supplementary material for: Predictive Modeling of Drug Response in Non-Hodgkin’s Lymphoma
Source: PLoS One. 2015 Jun 10;10(6):e0129433. doi: 10.1371/journal.pone.0129433 (PMC4464754; doi:10.1371/journal.pone.0129433)
Supplement: S1 Text — (DOCX) [file pone.0129433.s002.docx]

**Predictive Modeling of Drug Response in Non-Hodgkin’s Lymphoma**

Hermann B. Frieboes,^*^ Bryan R. Smith, Zhihui Wang, Masakatsu Kotsuma, Ken Ito, Armin Day, Michael Loewenberg, Benjamin Cahill, Colin Flinders, Shannon Mumenthaler, Parag Mallick, Eman Simbawa, A.S. AL-Fhaid, S. R. Mahmoud, Sanjiv S. Gambhir,^*^ and Vittorio Cristini^*^

* Correspondence should be addressed to

H.B.F. (project coordination and experimental data analysis, [hbfrie01@louisville.edu](mailto:hbfrie01@louisville.edu));

S.S.G. (experiments, [sgambhir@stanford.edu](mailto:sgambhir@stanford.edu));

V.C. (mathematical modeling, [VCristini@salud.unm.edu](mailto:VCristini@salud.unm.edu)).

**Supplementary Material**

**Evaluation of IC50 with Mafosfamide**

We noted that the differential in IC50 between *Eµ-myc/Arf-/-* and *Eµ-myc/p53-/-* cells (~13×) observed with Doxorubicin at 48 hours was very different when these cells were treated with Mafosfamide, a chemotherapy agent currently undergoing clinical trials for treatment of Non-Hodgkin’s Lymphoma. In this case, the IC50 was 0.08 μM and 8 μM, respectively, a ~100× differential (data not shown).

**Immunohistochemistry**

Representative whole-tumor sections of *Eµ-myc/Arf-/-* and *Eµ-myc/p53-/-* tumors showing viable and necrotic cells (stained for H&E), hypoxia (stained for HIF-1α), and vascularization (stained for CD31) are shown in **S2** and **S3 Figs**. Vascularization staining for both tumor types showing typical vessel sizes is in **S3(C-D) Fig**. The figure also highlights the tighter packing of the drug-resistant *Eµ-myc/p53-/-* cells compared to the drug-sensitive *Eµ-myc/Arf-/-* in samples taken in the middle of the tumor (Set S3). Hypoxia staining was used to estimate the diffusion distance of oxygen by measuring the distance from hypoxic tissue to near regions stained for vasculature (CD31).

**Sensitivity Analysis**

In a previous local sensitivity analysis (LSA) study [[1](#_ENREF_1),[2](#_ENREF_2)], we used a sensitivity coefficient as an index to evaluate how a change in a model parameter affects the overall system response. This coefficient is calculated by the following equation:

$S_{p}^{M}=\frac{(M_{i}-M_{0})/M_{0}}{(p_{i}-p_{0})/p_{0}},$ (1)

where *p* represents the parameter that is varied and *M,* the response of the system; *M_0_* is obtained by setting all parameters to their reference (unperturbed) values, and thus (*M_i_-M_0_*) is the change in *M* due to the change in *p*, i.e., (*p_i_-p_0_*). In our case, the system response *M* corresponds to the fraction of tumor volume killed $f_{\text{kill}}$, and *p* corresponds to one of the three parameters ($L$, $r_{\text{b}}$, and $\text{BVF}$) under consideration.

For each parameter, we created 101 variations, through a range of +/-50% of the parameter’s reference value and with a 1.0% variation interval. A 50% variation has been assumed as reasonable in molecular pathway analysis [[3](#_ENREF_3)]. The reference parameter values are summarized in **S1 Table**. Only one parameter was varied at a time, and all other parameters were held fixed at their reference values. **Fig. 6** illustrates the analysis results. For both drug-sensitive and drug-resistant cell lines, $\left|S\right|$ for *L* increases with variation change, whereas $\left|S\right|$ for $r_{\text{b}}$ and BVF decreases with variation change. As a result, the peak maximum of $\left|S\right|$ plots of *L* occurs at a variation of 1.5-fold of its reference value, and the peak maxima of $\left|S\right|$ plots of $r_{\text{b}}$ and BVF occur at a variation of 0.5-fold of their reference values (see **S1 Table**). BVF appears to be the most sensitive parameter for both cell lines.

**References**

1. Wang Z, Birch CM, Deisboeck TS. Cross-scale sensitivity analysis of a non-small cell lung cancer model: linking molecular signaling properties to cellular behavior. Biosystems 2008;92:249-58.

2. Wang Z, Birch CM, Sagotsky J, Deisboeck TS Cross-scale, cross-pathway evaluation using an agent-based non-small cell lung cancer model. Bioinformatics 2009;25:2389-96.

3. Tasseff R, Nayak S, Salim S, Kaushik P, Rizvi N, Varner JD. Analysis of the molecular networks in androgen dependent and independent prostate cancer revealed fragile and robust subsystems. PLoS One 2010;5:e8864.
