# Supplementary figures and images for: Predictive Modeling of Drug Response in Non-Hodgkin’s Lymphoma
Source: PLoS One. 2015 Jun 10;10(6):e0129433. doi: 10.1371/journal.pone.0129433 (PMC4464754; doi:10.1371/journal.pone.0129433)

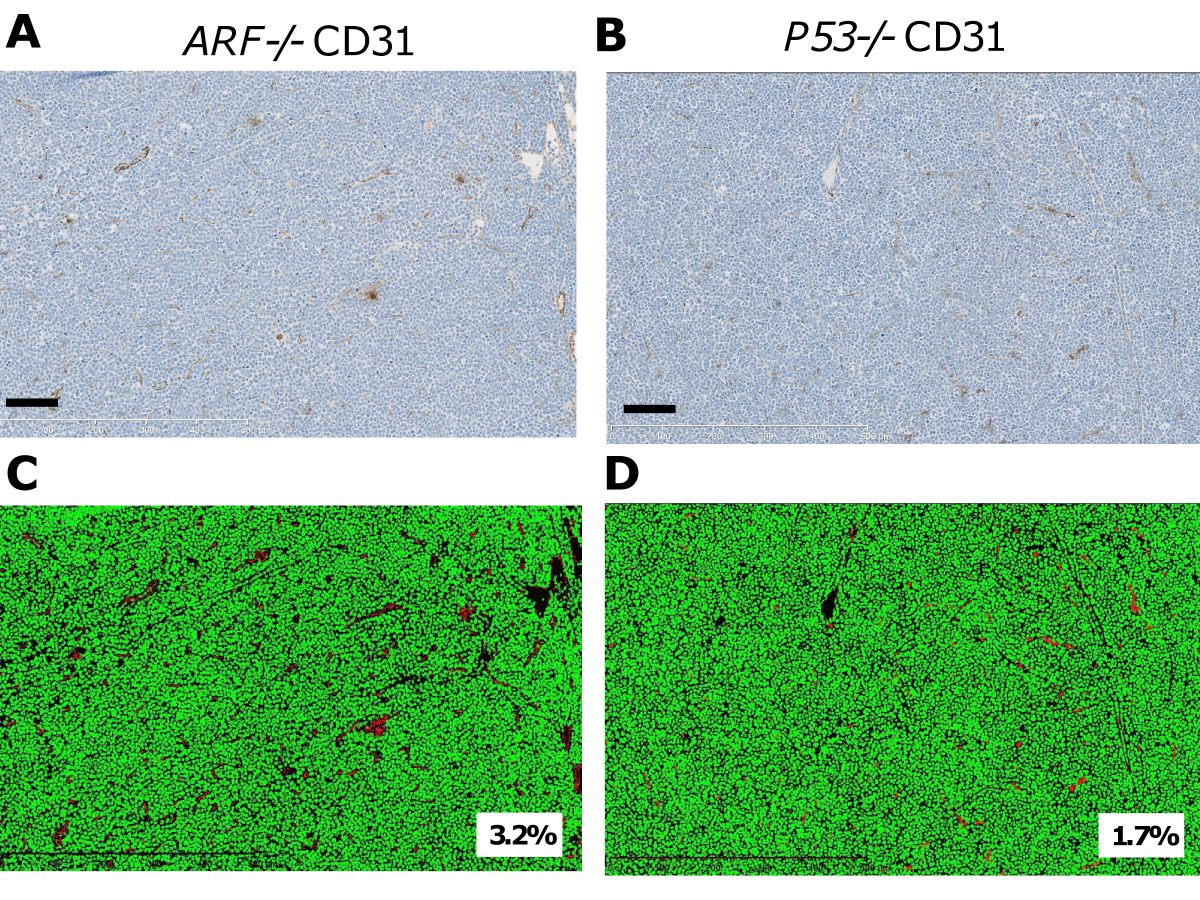

Supplement: S1 Fig — The process is illustrated through the quantification of CD31 staining in Set S3 in the center of the tumor reflecting blood volume fraction for Eμ-myc Arf-/- (drug-sensitive) and Eμ-myc p53-/- (drug-resistant) lymphoma cells. Positive staining shown in panels A and B is converted to red and negative staining to green in panels C and D to obtain a quantitative measure of apoptotic activity, as calculated in the text. Results are shown in bottom right insets. (TIF) [file pone.0129433.s003.tif]

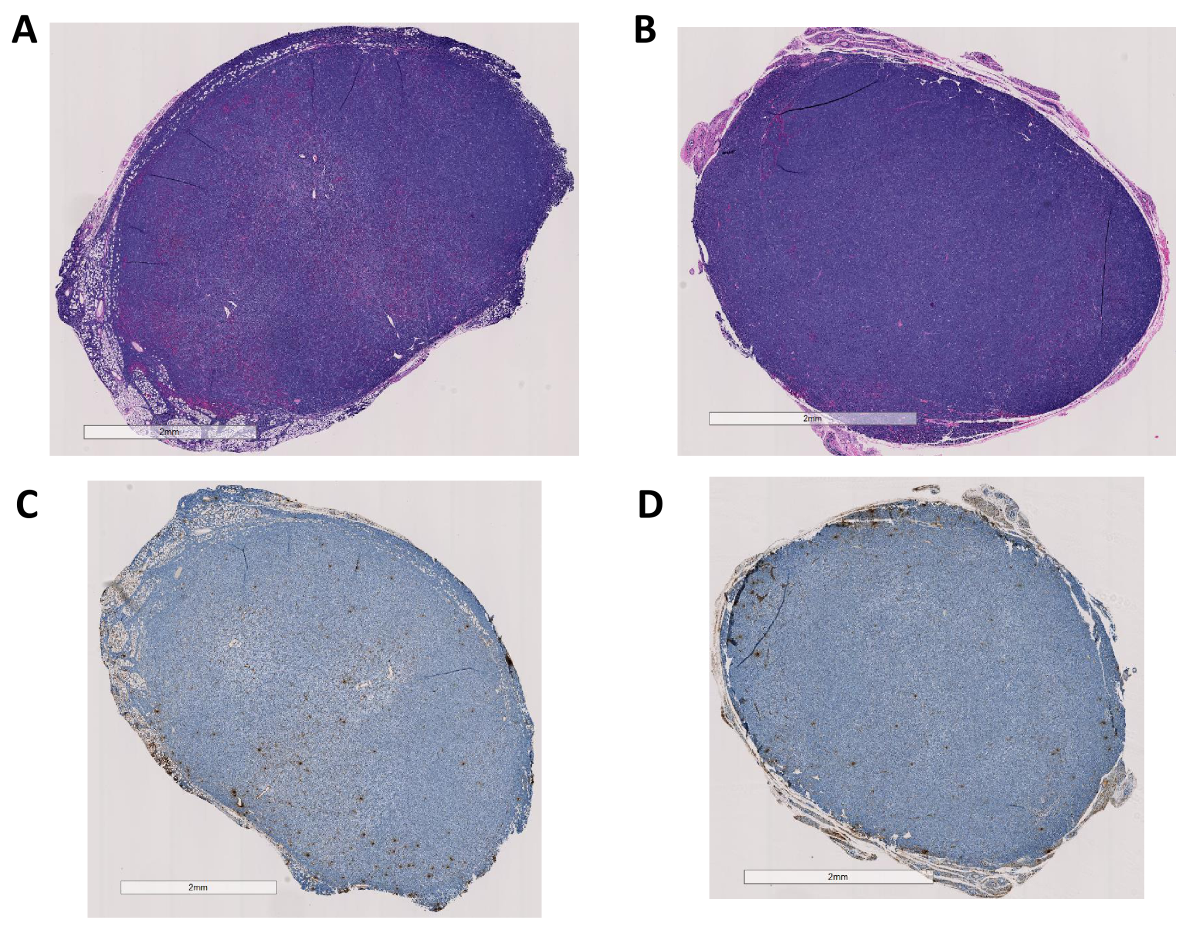

Supplement: S2 Fig — Representative whole-tumor histology sections for Eμ-myc/Arf-/- (left column) and Eμ-myc/p53-/- (right column) tumors, showing viable and necrotic cells (stained for H&E, panels A and B) and hypoxia (stained for HIF-1α, panels C and D, brown color) in the middle of the tumor (Set S3). Bar, 2 mm. (TIF) [file pone.0129433.s004.tif]

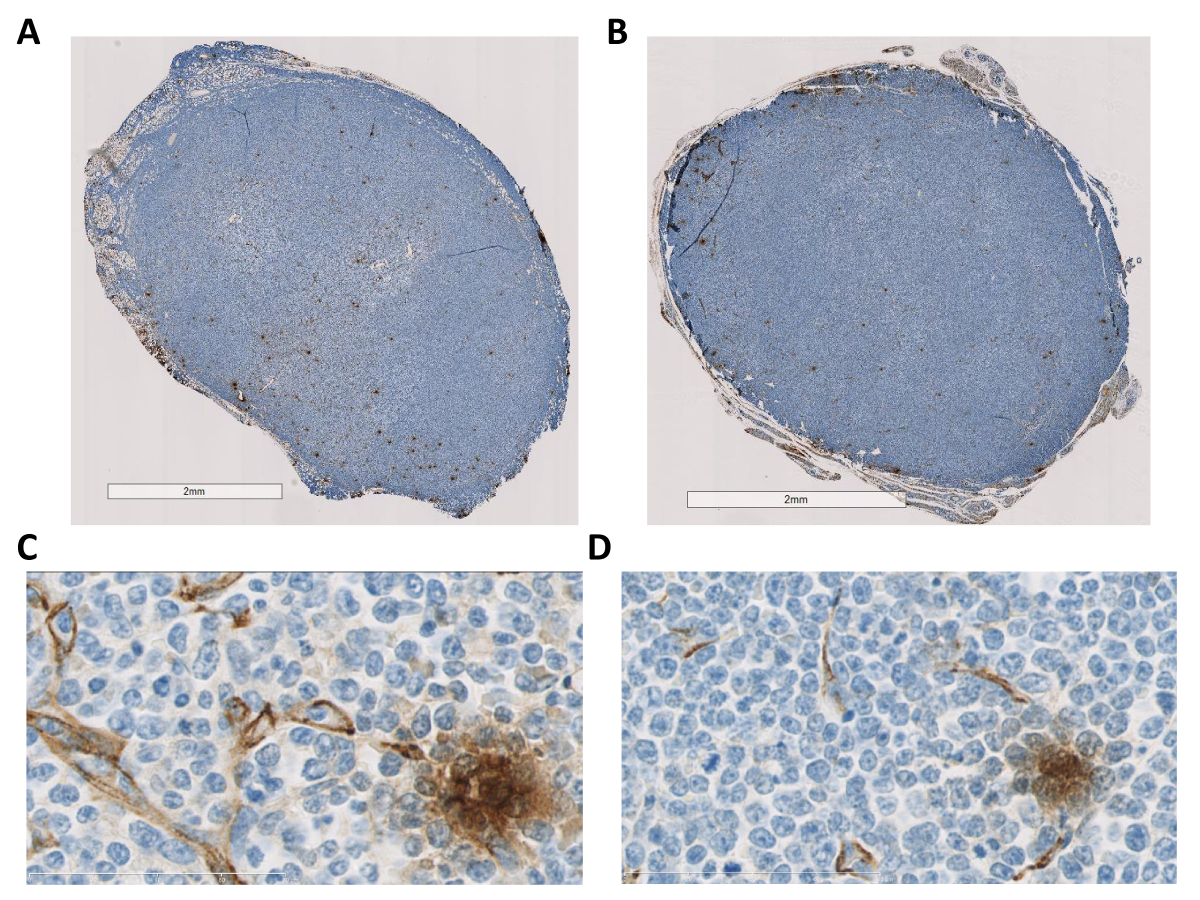

Supplement: S3 Fig — Representative whole-tumor vascularization (CD31) staining for Eμ-myc/Arf-/- (A) and Eμ-myc/p53-/- (B) tumors. Higher magnification (100x) images (C & D) show corresponding typical vessels (brown color) (100x). Capillaries are thinner elongated structures while veins are larger. The tighter packing of the drug-resistant Eμ-myc/p53-/- cells compared to the drug-sensitive Eμ-myc/Arf-/- can be visually appreciated in these samples taken in the middle of the tumor (Set S3). (40x). (TIF) [file pone.0129433.s005.tif]

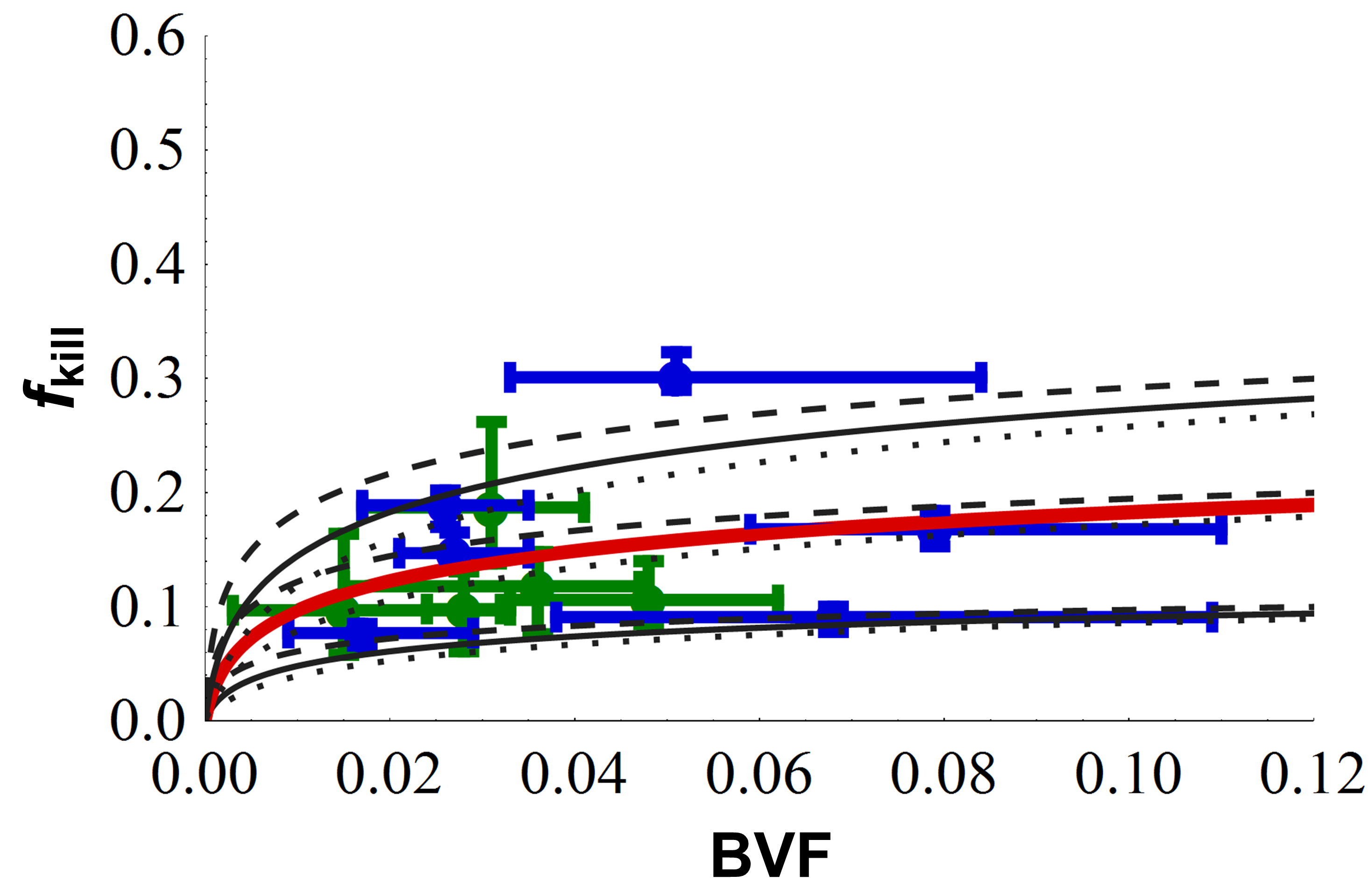

Supplement: S4 Fig — The bold solid line in red represents the best fit case determined in Fig 5. Color scheme: {fkillM = [0.5, 1.0, 1.5]-fold of its best fit} = {gray, red, gray}; green for drug sensitive cell line (Eμ-myc/Arf-/-) and blue for drug resistant cell line (Eμ-myc/p53-/-). Line scheme: {r b / L = [0.5, 1.0, 1.5]-fold of its best fit} = {dashed, solid, dotted}. (TIF) [file pone.0129433.s006.tif]
